# Hepatic angiomyolipoma: differential diagnosis from other liver tumors in a special reference to vascular imaging - importance of early drainage vein

**DOI:** 10.1186/s40792-014-0008-y

**Published:** 2015-01-31

**Authors:** Masato Yoshioka, Go Watanabe, Hiroshi Uchinami, Kazuhiro Kudoh, Yuko Hiroshima, Toshiaki Yoshioka, Hiroshi Nanjo, Masato Funaoka, Yuzo Yamamoto

**Affiliations:** Department of Gastroenterological Surgery, Akita University Graduate School of Medicine, 1-1-1 Hondo, Akita, 010-8543 Japan; Department of Pathology, Akita University School of Medicine, Akita, 010-8543 Japan; Department of Internal Medicine, Yokote Municipal Hospital, 5-31 Negishi-cho, Yokote, Akita 013-8602 Japan

**Keywords:** Angiomyolipoma, Liver, Hepatocellular carcinoma, Early drainage vein, Angiography

## Abstract

A 51-year-old female had been diagnosed with a hemangioma in the hepatic segment 6 (S6). After a 6-year follow-up, enlargement of the tumor was detected. The tumor was clearly enhanced in the arterial phase, and the enhancement remained in the portal phase on computed tomography (CT). Although the primary differential diagnosis on CT was hepatocellular carcinoma (HCC), we worried about the possibility of other vessel system tumors because the tumor remained to be enhanced at the portal phase for HCC and all tumor markers of HCC were negative. We performed angiography to determine the tumor nature and to seek other tumors. Angiography showed tumor stain at the hepatic S6 with an early obvious drainage vein from the tumor flowing through the right hepatic vein into the inferior vena cava. In addition to tumor stain and the drainage vein, there were many small poolings of contrast medium in the whole liver, which were suspected as dilatation of the hepatic peripheral artery. We suspected the tumor as a benign tumor such as hepatocellular adenoma or focal nodular hyperplasia, but the possibility of HCC could not be ruled out. Hepatic posterior sectionectomy was done to completely remove the drainage vein with the tumor. Intraoperative histological examination revealed the tumor as not malignant and not HCC. Later, immunohistochemical analysis uncovered that the tumor had high expression of HMB-45 and, therefore, the final diagnosis was angiomyolipoma. We think that detecting an early drainage vein from the tumor would be a key point for diagnosing hepatic angiomyolipoma.

## Background

Angiomyolipoma (AML) is a rare benign mesenchymal neoplasm most often arising in the kidney, and extrarenal AMLs are uncommon. Among these extrarenal sites, the liver is the most common site [1]. The appearance in other sites is extremely rare [2,3]. AML is composed of heterogeneous mixtures of smooth muscle cells, mature adipose tissues, thick-walled blood vessels, with occasional foci of extramedullary hematopoiesis [4-8]. Histological diagnosis is performed by immunohistochemical staining against melanoma-associated antigen (HMB-45) [9]. However, hepatic angiomyolipoma (HAML) has usually been misdiagnosed as hepatocellular carcinoma (HCC) with a frequency of more than 50% due to significant overlap of the imaging features [10-13]. Differential diagnosis between HAML and HCC is very important because their prognosis is strikingly different. In some reports, early venous drainage from the tumor is a useful feature for differentiating HAML from HCC [14-18]. If early venous drainage from the tumor is detected preoperatively, it would be helpful for diagnosing HAML.

We here report a case of HAML showing an early venous drainage from the tumor to the hepatic vein detected by angiography preoperatively and review the reported cases having early venous drainage in a special reference to the early drainage veins and its detection modalities.

## Case presentation

**Figure 1:**
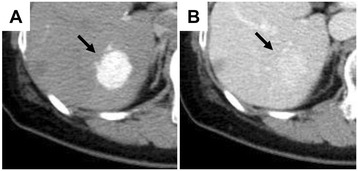
**Computed tomography (CT) imaging of the abdomen. A**. There is a tumor at the segment 6 of the liver, which is well enhanced by contrast medium at the arterial phase (*arrow*). **B**. The tumor remains to be enhanced by contrast medium in portal phase (*arrow*).

**Figure 2:**
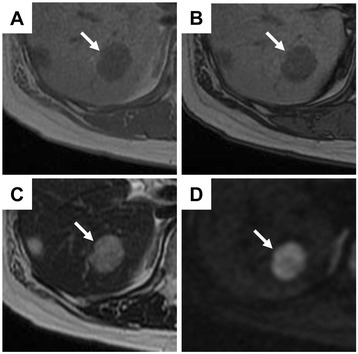
**Magnetic resonance imaging (MRI). A**. In-phase image of chemical shift T1-weighted MRI shows low intensity at the tumor of segment 6 (*arrow*). **B**. No further decrease of signal intensity was observed in out-of-phase image (*arrow*). **C**. T2-weighted MRI shows high intensity (*arrow*). **D**. Diffusion-weighted imaging showed high intensity at the tumor (*arrow*).

A 51-year-old female had a medical checkup by a neighboring hospital, and ultrasonography (US) detected a tumor of 9.5 mm in diameter in the posterior segment 6 (S6) of the liver. Abdominal computed tomography (CT) detected a tumor at S6, and it had been diagnosed as hemangioma. She was subsequently followed up by CT every year. During the 4 years after the first diagnosis, although the tumor was slightly enlarged to 20 mm in diameter, the diagnosis by US and CT was still hemangioma. However, in the next 2 years, the tumor growth was accelerated and had grown to 30 mm in diameter. Furthermore, on dynamic CT image, the tumor was well enhanced by contrast medium in the arterial phase (Figure 1A) and remained to be enhanced in the portal phase (Figure 1B). In-phase image of chemical shift T1-weighted MRI showed low intensity at the tumor of S6 (Figure 2A). No further decrease of signal intensity was observed in out-of-phase image (Figure 2B). T2-weighted MRI showed high intensity (Figure 2C) at the tumor. These findings suggested less possibility that the tumor had fat component as usual in AML. Diffusion-weighted imaging (DWI) showed high intensity at the tumor (Figure 2D) and apparent diffusion coefficient value was 1.57 × 10^−3^ mm^2^/s; this value was lower than that for AML (1.92 × 10^−3^ mm^2^/s) and higher than that for HCC (1.33 × 10^−3^ mm^2^/s) [19].

From these imagings by CT and MRI, the tumor was rather suspected as HCC and introduced to Akita University Hospital. In her past medical history, she had idiopathic thrombocytopenic purpura in pregnancy, but it was cured without treatment. On admission, blood examination showed slight anemia and decrease of white blood cell count (Table 1). She was negative for hepatitis B surface antigen and hepatitis C antibody. All serum tumor markers of alpha-fetoprotein (AFP), AFP-L3, protein induced by vitamin K absence or antagonist-II, carcinoembryonic antigen, and carbohydrate antigen 19-9 were within normal limits. The indocyanine green dye relation rate after 15 min was 2.1%, in the normal range.

**Table 1:** **Laboratory data on admission**

WBC	2,200/mm^3^	ChE	338 IU/L
RBC	405 × 10^4^/mm^3^	Na	142 mEq/L
Hb	12.7 g/dL	K	4.0 mEq/L
Ht	38.3%	Cl	103 mEq/L
Plt	16.6 × 10^4^/mm^3^	Ca	9.6 mg/dL
AST	17 IU/L	CRP	0.02 mg/dL
ALT	14 IU/L	Glucose	88 mg/dL
ALP	223 IU/L	HbA1c	5.1%
LDH	144 IU/L	CEA	2.6 ng/mL
γ-GTP	30 IU/L	CA19-9	17.4 U/mL
T-Bil	0.8 mg/dL	AFP	3.3 ng/mL
TP	7.0 g/dL	AFP-L3	0.5%
Alb	4.5 g/dL	PIVKA-II	15 mAU/mL
BUN	13.5 mg/dL	HBS antigen	(−)
Cre	0.52 mg/dL	HCV antibody	(−)
AMY	332 IU/L		

WBC, white blood cell; RBC, red blood cell; Hb, hemoglobin; Ht, hematocrit; Plt, platelet; AST, aspartate aminotransferase; ALT, alanine aminotransferase; ALP, alkaline phosphatase; LDH, lactic dehydrogenase; γ-GTP, gamma glutamyl transpeptidase; T. Bil, total bilirubin; TP, total protein; Alb, albumin; BUN, blood urea nitrogen; Cr, creatinine; AMY, amylase; CHE, cholinesterase; Na, sodium; K, potassium; Cl, chloride; Ca, calcium; CRP, C-reactive protein; HbA1c, hemoglobin A1c; CEA, carcinoembryonic antigen; CA19-9, carbohydrate antigen 19-9; AFP, alpha-fetoprotein; AFP-L3, alpha-fetoprotein fraction L3; PIVKA-II, protein induced by vitamin K absence or antagonist-II; HBs antigen, hepatitis B surface antigen; HCV antibody, hepatitis C virus antibody.

**Figure 3:**
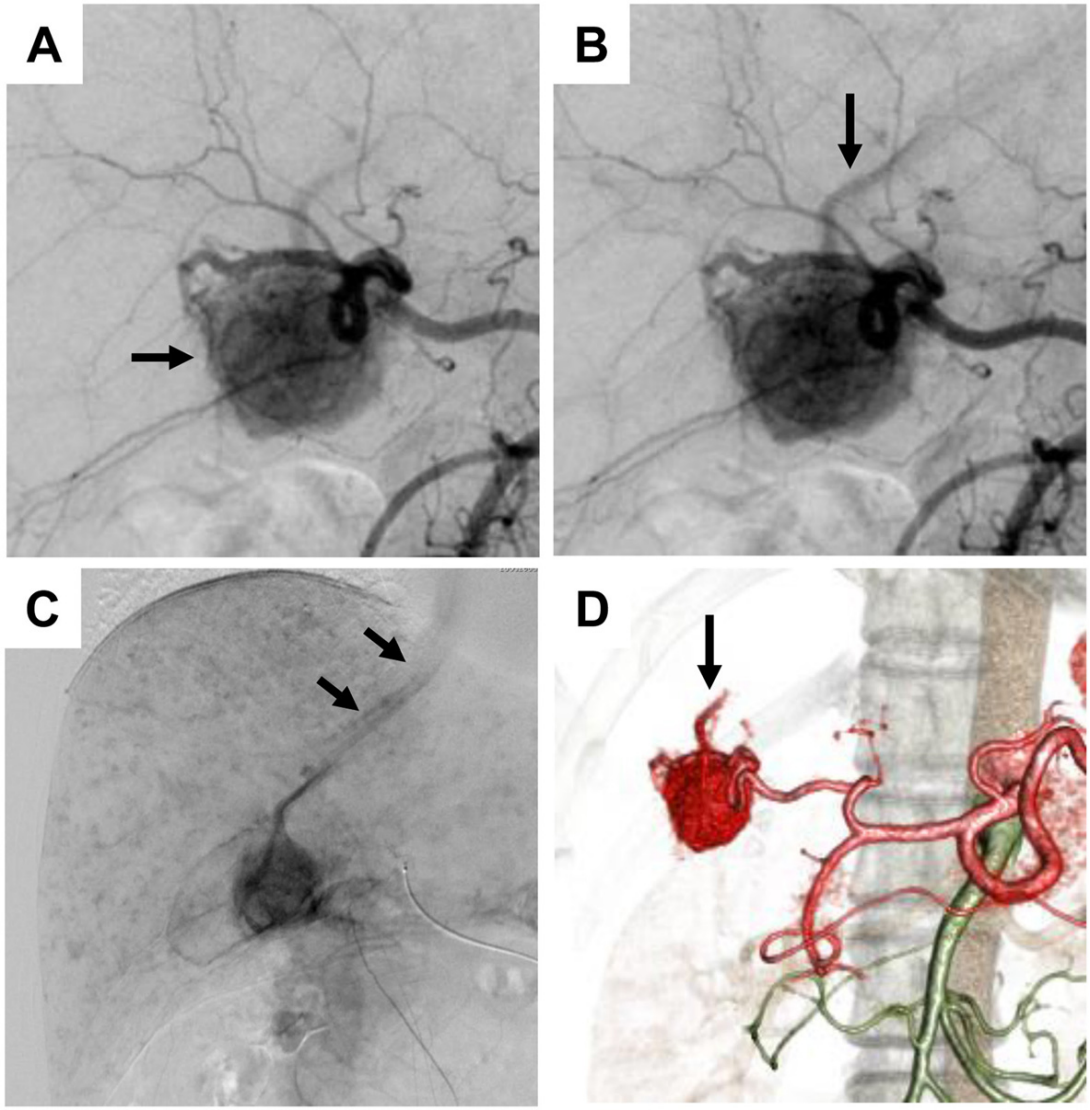
**Digital subtraction angiography (DSA) and reconstructed image of computed tomography during angiography. A**. Angiography shows a tumor stain (*arrow*) at the hepatic segment 6 with bold feeding artery. An early drainage vein from the tumor is detected at hepatic arterial phase. **B**. Blood flow of drainage vein is gradually clear (*arrow*). **C**. Contrast medium flows intensely into inferior vena cava (*arrows*). There were many small poolings of enhanced medium, which are suspected as peripheral blood dilatation, around the whole liver. **D**. Three dimension reconstructed image of computed tomography during arterial angiography. There is an efferent vessel from the tumor (*arrow*).

We worried about the differential diagnosis between HCC and other vessel system tumors because the tumor remained to be enhanced at the portal phase for HCC and all tumor markers of HCC were negative. To determine the nature of the tumor and to seek other tumors, we performed positron emission tomography (PET) imaging using an ^18^ F-fluorodeoxyglucose (FDG) tracer and digital subtraction abdominal angiography (DSA). There was no hot accumulation of FDG to the tumor, and the lesion was rather cold. DSA showed a tumor stain at the hepatic S6 (Figure 3A). We detected further an early drainage vein from the tumor flowing through the right hepatic vein into the inferior vena cava (IVC) (Figure 3A,B,C). In addition to tumor stain and drainage vein from the tumor, there were scattered many small poolings of contrast medium in the whole liver, which were suspected as dilatation of the hepatic peripheral artery (Figure 3C). Figure 3D shows the drainage vein from the tumor in the image of CT during arterial angiography. Because of the reason of the strange pattern of poolings of contrast medium in the tumor, we suspected the tumor as a benign tumor such as hepatocellular adenoma or focal nodular hyperplasia although the possibility of HCC could not be ruled out yet. Although the preoperative biopsy was a diagnostic method of choice, she did not desire it because of the complication risk and the possible surgery when the result was ambiguous. Furthermore, we had an experience of an atypical liver hemangioma - the present patient was also followed up for 6 years with a diagnosis of hemangioma - with an arteriovenous shunt in which the patient developed heart failure due to massive cardiac load [20].

**Figure 4:**
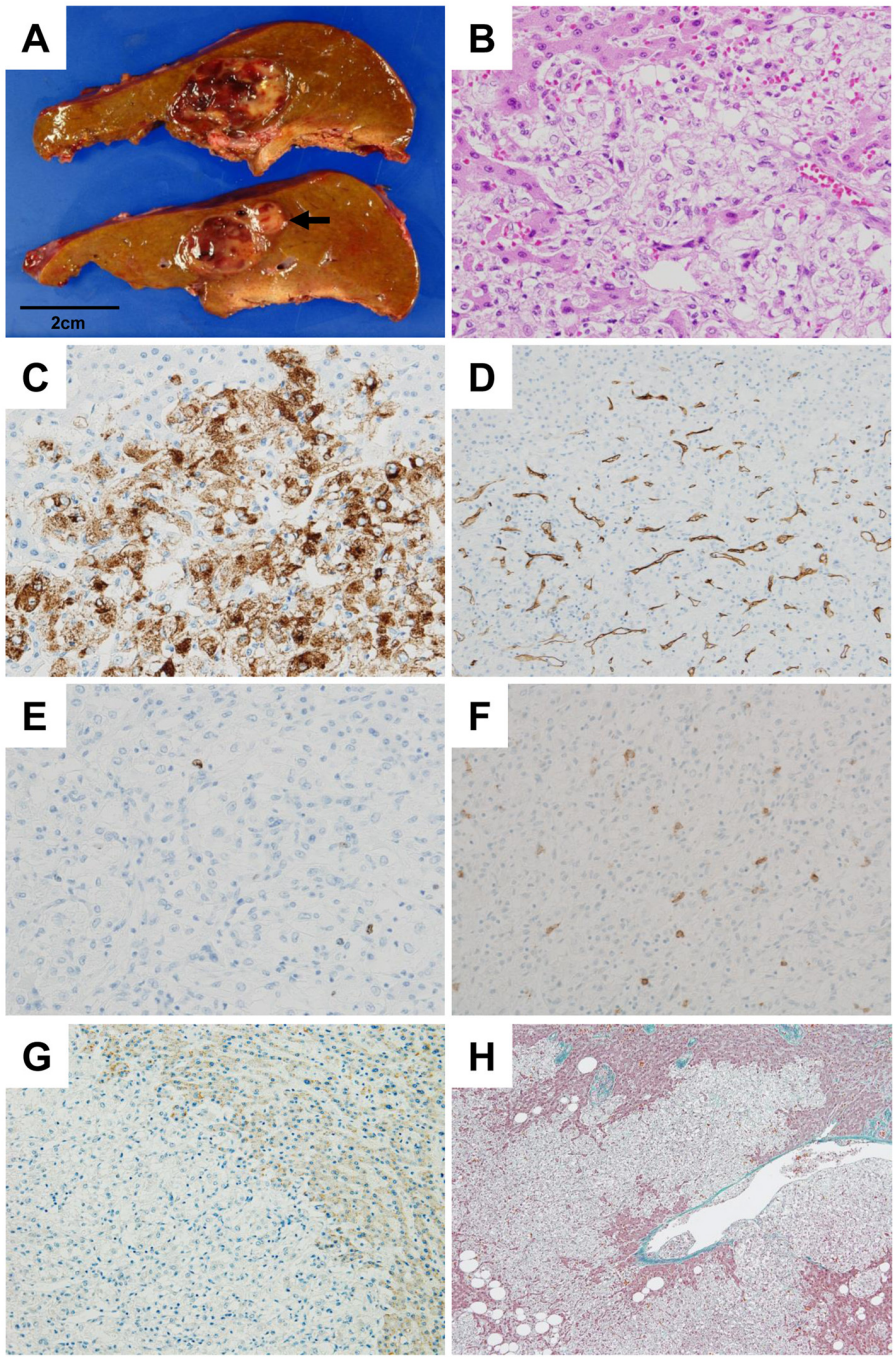
**Gross and histological findings. A**. The resected fresh specimen showed dark yellowish soft tumor, measuring 31 mm in diameter, with no capsule, and a small amount bleeding on the cutting surface. A neighboring small mass of 6.5 mm in diameter existed (*arrow*). **B**. Histological examination revealed that the tumor showed that there were many epithelioid myoid cells, epithelioid clear cells with sinusoidal vessels, and mature adipose tissues (H & E staining, objective; ×40). **C**. Immunohistochemical analysis revealed that epithelioid myoid cells showed high expression of HMB-45 (objective; ×40). **D**. Many capillaries positive for CD34 antigen had grown (objective; ×20). **E**. The Ki67 index was 0.3% (objective; ×40). **F**. The tumor cells were negative for c-kit (CD117), and only plasma cells were positive (objective; ×20). **G**. Expression of TSC1/hamartin reduced in comparison with surrounding liver tissue (tumor; left lower, normal liver; right upper, objective; ×20). **H**. Elastica-Masson stain. Tumor infiltrated into hepatic veins (objective; ×4).

We performed posterior sectionectomy by a Glissonean pedicle transection method for several reasons: the tumor location was near the Glissonean bifurcation to S6 and S7, the volume of S7 was relatively small, and to remove the abnormal drainage vessel which runs between the S6 and S7. The liver looked normal macroscopically and neither other tumors nor small hemangiomas were recognized in the liver except the known simple liver cyst. We served several drainage vessels into the right hepatic vein, but we could not clearly specify which was the early drainage vein of discussion. Interruption of feeding inflow to the tumor by Glissonean pedicle transection might make the drainage vein inconspicuous. Intraoperative histological examination revealed that the tumor was not malignant and not HCC. The resected fresh specimen showed dark yellowish soft tumor, measuring 31 mm in diameter, with no capsule, and a small amount bleeding on the cutting surface. There existed a neighboring small mass of 6.5 mm in diameter (Figure 4A). Histological examination showed that the tumor consisted of many epithelioid myoid cells, epithelioid clear cells with sinusoidal vessels, and mature adipose tissues (Figure 4B). A distinctive capsule did not exist, and the tumor infiltrated into surrounding normal liver tissues. Immunohistochemical analysis uncovered that the epithelioid myoid cells expressed HMB-45 strongly (Figure 4C). Immunoreactive proteins for cytokeratin AE1/AE3, AFP, desmin, S100 protein, and CD68 were all negative. Many capillaries positive for CD34 antigen has grown (Figure 4D). The Ki67 index was only 0.3% (Figure 4E), and tumor cells were negative for c-kit (CD117) (Figure 4F). Expression of tuberous sclerosis complex (TSC) 1/hamartin, a protein encoded by the *TSC1* gene, was reduced in the tumor in comparison to the surrounding liver tissue (Figure 4G). Final diagnosis was AML. The tumor infiltrated into the hepatic veins (Figure 4H). There were neither other tumors nor hemangioma in the surrounding normal liver tissue, where many small poolings of contrast medium were detected on angiography. Nineteen months had passed after surgery; no new lesions were detected on CT.

## Discussion

In the literature describing the preoperative diagnosis of HAML [10,13,21-25], the findings are as follows: (1) hypervascular imaging; (2) fatty component on CT, MRI, and US, which shows hyperechoic lesion on US, low attenuation with a density of less than −20 Hounsfield units on CT, and hyperintensity on both T1- and T2-weighted sequences of MRI; (3) positive actin and HMB-45 stains on biopsy specimen. However, differential diagnosis of HAML from other fat-containing lesions of the liver, especially HCC with fatty metamorphosis, is very difficult [13]. The proportion of fatty tissue in HAML varies greatly, ranging from 5% to 90% [24]. This variety would influence the images of MRI. In the present case, both in-phase and out-of-phase images showed similar low intensity, probably due to non-rich fat component and made our diagnosis shift to other tumors than HAML. In fact, misdiagnosis occurs at a frequency of more than 50% due to a significant overlap of the features of imaging in HAML and HCC [10-13]. Other tumors abounding with blood vessels, such as hemangioma, focal nodular hyperplasia, or hepatic adenoma, showing resembling imaging features, are also difficult in differential diagnosis.

Unlike these similar imaging features, a presence of an early drainage vein has been recently focused in some reports. In the present patient, we could observe an extra-tumoral streak drainage vein toward the right hepatic vein from a thick enhanced tumor with clear margin in DSA and an arterial phase of dynamic MDCT. To the best of our knowledge, in English literature so far, at least 14 patients of HAML have been reported to have early drainage veins connecting to hepatic veins (Table 2) [14-17,19,26]. Iwao et al. reported five cases of HAML, and four patients (80%) had an early drainage vein flowing to the hepatic vein [26]. Jeon et al. reported the study of the presence of efferent vessels between fat-deficient HAML (ten patients) and HCC (29 patients) using triple-phase dynamic CT or multi-detector computed tomography (MDCT) [17]. In their report, an early drainage vein that flows into the hepatic vein was considered to be positive in 50% with HAML. On the other hand, in HCC patients, it was only 7%. Although the report of Wang et al. might contaminate patients with arterioportal (AP) shunting in their series, they reported similar percentages - 16 of 22 for HAMLs (72.7%) (obviously or at least faintly) and 2 of 25 in HCCs (8%). Compared to HAML, the drainage vein of HCC was faint and negligible. In HAML, an early drainage vein was present even in a small tumor, the size of which was 2 cm in diameter. Meanwhile, the size of HCCs having a drainage vein was over 12 cm in diameter. It means that in comparison with HCC, the drainage vein would be found in a considerable number of HAMLs while the tumor size is still small. Thus, the presence of early drainage vein is anticipated as a useful feature for distinguishing HAML from HCC.

**Table 2:** **Reported cases having early drainage vein in hepatic angiomyolipoma**

**Author**	**Publish year**	**Number of cases**	**Detection modality**	**Drainage vein**
1. Murakami [14]	1993	1	DSA	Hepatic vein
2. Yoshimura [15]	2002	2	DSA	Hepatic vein
3. Zheng [16]	2005	2	DSA, CED, US	Hepatic vein
4. Jeon [17]	2010	5	MDCT	Hepatic vein
5. Iwao [26]	2014	4	DSA	Hepatic vein
6. Wang [19]	2014	16	MRI	*Not described* ^a^
7. Present case		1	DSA	Hepatic vein

DSA, digital subtraction angiography; CED, contrast-enhanced Doppler; US, ultrasound; MDCT, multi-detector computed tomography; MRI, magnetic resonance imaging,

^a^Drainage to hepatic vein, inferior vena cava, or portal vein.

Here, we must be careful for an AP shunting that is often seen in angiogenic liver tumors. The AP shunts are reportedly rather frequently found in hemangiomas as well, especially in that with rapid enhancement [27]. They are, however, usually depicted as a wedge-shaped or irregularly shaped parenchymal fuzzy enhancement adjacent to the tumor in the hepatic arterial phase of dynamic CT. This CT feature is different from that of the early drainage vein discussed above and may be useful to distinguish them. Although rare, there are some reports of HAMLs with occasional association of AP shunts, too (one case reported by Saito et al. [18] and three cases by Jeon et al. [17]). But it is necessary to differentiate AP shunts from the early drainage vein. Concerning the detection modality for an early drainage vein, the usefulness of dynamic MDCT is reported [17,18]. In our case, we first noticed the drainage vein during angiography, but careful image reading of dynamic MDCT could confirm it.

Regarding the multiple small poolings of contrast medium scattered in the whole liver during angiography in our patient, the similar phenomenon has been reported [16]. The lesions of the case report were diagnosed as multiple hemangiomas by surgery, but in our patient, the liver looked normal macroscopically and no hemangiomas were detected in the resected specimen either macroscopically or histopathologically. In addition, we could not find any micro HAML or the lesion that might be tentatively expressed as a bud of HAML. Therefore, it may be too hasty to conclude that these multiple small poolings of contrast medium are indubitably small hemangiomas. AML occurs frequently in the kidney, where it is closely related to tuberous sclerosis [28,29]. Tuberous sclerosis is an autosomal dominant genetic disorder that arises from germ line mutations in *TSC1* or *TSC2*, which encode hamartin and tuberin, respectively [30,31]. In tuberous sclerosis patients, abnormality in blood vessels occasionally happens, such as aneurysms or narrowing of blood vessels. Therefore, we examined the expression of TSC1/hamartin in the tumor in the protein level immunohistochemically to address the mystery of the multiple small poolings of contrast medium. The expression of TSC1/hamartin was reduced in the tumor tissue in comparison with the surrounding liver tissue. This result might show a possible relationship of the HAML tumor with decreased TSC1/hamartin expression in this patient but did not answer the abnormal poolings of contrast medium in the surrounding liver tissue during angiography. Close follow-up of the patient for the recurrence of nodules is required, and the reproducibility of the same abnormal poolings in future imagings is intriguing.

Lastly, there are some reports of malignant HAML. Deng et al. reported malignant characteristics as marked cell atypia, vascular invasion, high proliferation activity (Ki67 index above 30%), and large tumor size [32]. In the present case, precise pathological examination revealed a tumor infiltration into the hepatic vein although the tumor was negative for FDG-PET and low in the Ki67 index. It is uncertain whether this finding of vascular invasion directly suggests the malignant transformation of this tumor or not [33], but preoperative detection of such a minute finding is dubious even by means of biopsy. Conservative management of the tumor that is diagnosed as HAML should be careful and will be recommended only in the condition of close follow-up, otherwise surgical resection is recommended.

## Conclusions

We presented a case of HAML with early drainage vein flowing to the hepatic vein. We think that detecting an early drainage vein would be a key to diagnose the HAML. In case an obvious drainage vein is detected, HALM will come to the very front line of differential diagnosis.

## Consent

Written informed consent was obtained from the patient for publication of this case report and any accompanying images. A copy of the written consent is available for review by the Editor-in-Chief of this journal.
